# Membrane Invaginations Reveal Cortical Sites that Pull on Mitotic Spindles in One-Cell *C. elegans* Embryos

**DOI:** 10.1371/journal.pone.0012301

**Published:** 2010-08-20

**Authors:** Stefanie Redemann, Jacques Pecreaux, Nathan W. Goehring, Khaled Khairy, Ernst H. K. Stelzer, Anthony A. Hyman, Jonathon Howard

**Affiliations:** 1 Max Planck Institute of Molecular Cell Biology and Genetics, Dresden, Germany; 2 European Molecular Biology Laboratory (EMBL), Cell Biology and Biophysics Unit, Heidelberg, Germany; Virginia Tech, United States of America

## Abstract

Asymmetric positioning of the mitotic spindle in *C. elegans* embryos is mediated by force-generating complexes that are anchored at the plasma membrane and that pull on microtubules growing out from the spindle poles. Although asymmetric distribution of the force generators is thought to underlie asymmetric positioning of the spindle, the number and location of the force generators has not been well defined. In particular, it has not been possible to visualize individual force generating events at the cortex. We discovered that perturbation of the acto-myosin cortex leads to the formation of long membrane invaginations that are pulled from the plasma membrane toward the spindle poles. Several lines of evidence show that the invaginations, which also occur in unperturbed embryos though at lower frequency, are pulled by the same force generators responsible for spindle positioning. Thus, the invaginations serve as a tool to localize the sites of force generation at the cortex and allow us to estimate a lower limit on the number of cortical force generators within the cell.

## Introduction

The positioning of the mitotic spindle is thought to be mediated by the interaction between astral microtubules, which grow outward from the spindle poles, and the plasma membrane [1], [2]. In some cases, such as the centering of the spindle in fission yeast, spindle movement relies on pushing forces that arise as growing astral microtubules push against the cortex [3], [4]. In other cases, such as during asymmetric cell division in budding yeast and in the one-cell *C. elegans* embryo, spindle movement away from the cell center relies on force-generating complexes located at the cortex that pull on astral microtubules contacting the cell cortex [5], [6]. Although the existence of pulling forces is well established by laser-cutting experiments [7], [8], and genetic and RNAi experiments have provided insight into the molecules necessary for force generation [9], individual force generation events have not been observed *in vivo*. Therefore, direct evidence for the location and number of the force generating elements is missing.

The translation of microtubule pulling forces into spindle movement presumably requires the microtubule end to be anchored to a relatively stiff platform at the plasma membrane, and it has been suggested that the acto-myosin cortex could provide such a platform [2], [10], [11]. Here we show that in *C. elegans* embryos possessing a weakened acto-myosin cortex, microtubule pulling forces give rise to invaginations of the membrane. This allows the direct visualization of individual force generation events in live embryos for the first time.

## Results

To weaken the cortex, we subjected embryos to partial depletion of the non-muscle myosin, NMY-2, which is essential for normal contractile function of the acto-myosin cortex [12], [13]. Partial depletions were studied because full disruption of the acto-myosin cortex interferes with anterior-posterior polarity and leads to defects in asymmetric pulling forces [12], [13]. After RNAi against *nmy-2* for 22 to 24 hours, we found that embryos properly established PAR polarity (fig. S1) and spindle movement was normal (fig. S2A). Moreover, in these embryos the tension on the spindle, as assessed using laser ablation, was not significantly altered on the posterior side. We did find a small but statistically significant (*p = 0.01*) increase on the anterior side compared to non-treated embryos, which is consistent with previous observations [14], [15] (fig. S2B,C). These observations suggest that under such conditions, pulling forces can still be transmitted to the spindle.

**Figure 1:**
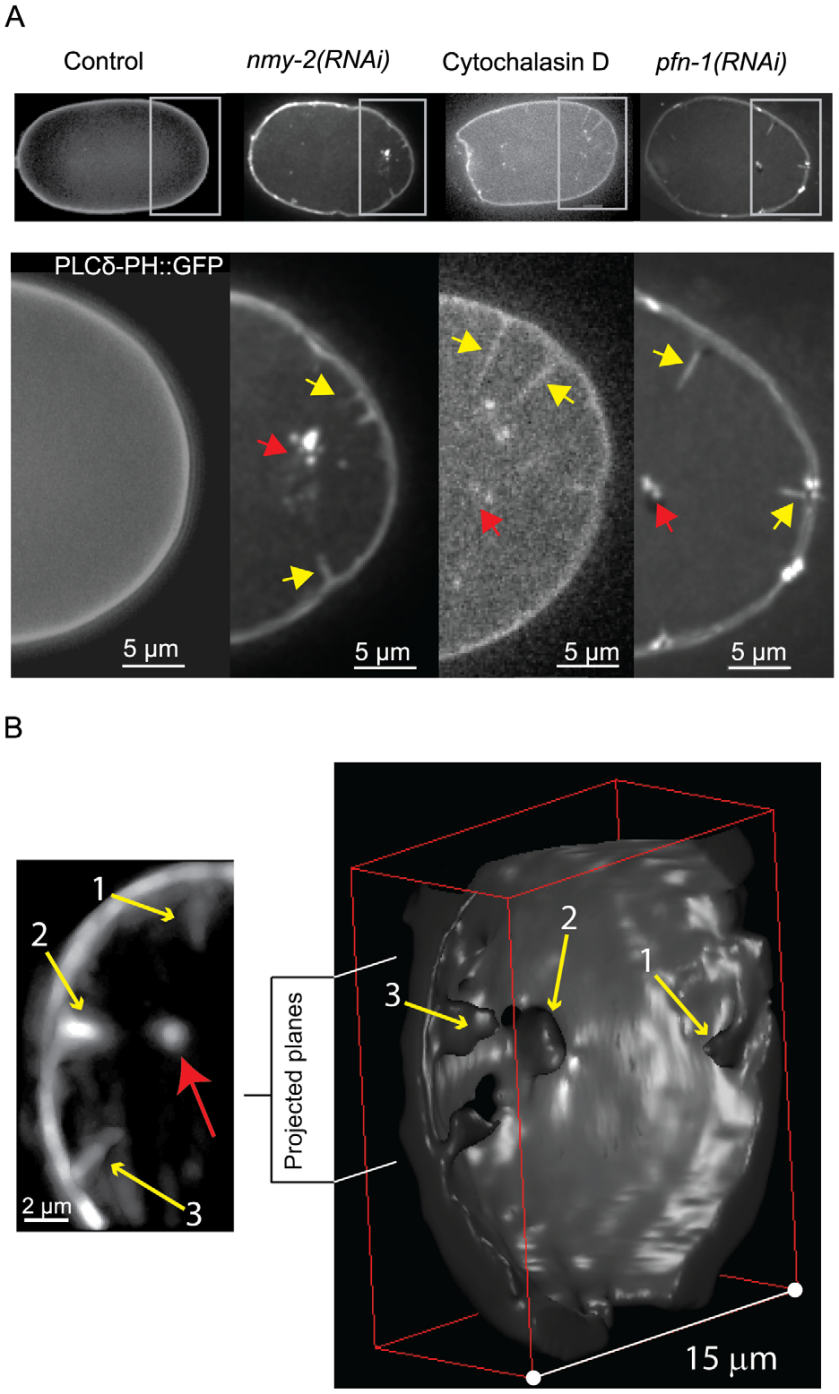
Invaginations imaged by spinning disc confocal and SPIM. **A**, Embryos expressed PH::GFP as a membrane marker were subject to the indicated treatment. Regions with invaginations, enclosed by rectangles, are enlarged below. Invaginations are marked by yellow arrows. Accumulation of the PH::GFP signal on the centrosomes occurs in all RNAi conditions and is indicated by red arrows. Scale bars are 5 μm. **B**, Selective plane illumination microscopy (SPIM) of a PH::GFP embryo treated with *nmy-2(RNAi)*. Left: z-projection of 10 slices of the indicated region of the 3D image. The original image was reduced to 8 bit and median filtered. Three invaginations (yellow arrows) all point toward the centrosome (red arrow). Scale bar is 2 μm. Right: Level-set segmentation and surface reconstruction of the region showing the conical basis (black hole) of the invaginations (yellow arrows) from the cytoplasmic side of the cell membrane.

Although pulling forces appeared normal, when we visualized the membrane in these embryos using the GFP-tagged PH-domain of phospholipase C δ 1, PLCδ1-PH::GFP [16], further referred to as PH::GFP, we noticed that the membrane was pulled into the cytoplasm, leading to invaginations that pointed inward toward the centrosomes (video S2, fig. 1A, compare first and second panels). Examination of untreated PH::GFP embryos revealed that invaginations also occur in embryos with a normal acto-myosin cortex (3.4±1.2 per embryo on the posterior pole, *n* = 5), but at a much lower frequency when compared to *nmy-2 (RNAi)* (42±6 on the posterior pole, *n* = 4 embryos). Errors are standard deviation (SD) unless otherwise noted. In both *nmy-2 (RNAi)* and untreated embryos, invaginations are enriched in the posterior. This observation and the fact that invaginations are directed away from the membrane toward the centrosome suggested to us that their appearance might be correlated with the microtubule-dependent pulling forces that drive posterior spindle displacement.

To gain insight into the nature of the invaginations, we examined them using Selective Plane Illumination Microscopy (SPIM) [17], which showed that the invaginations were continuous with the membrane at the cell periphery (fig. 1B). The bases of the invaginations were broad, typically greater than 2 µm in diameter and formed a cone about 1–2 µm deep (fig. 1B). This cone extended as a thin process toward the centrosome. The diameter of the process could not be resolved, suggesting that it was below 200 nm. This shape is similar to that of membrane tubules pulled from membrane vesicles in vitro [18], [19]. Together with the fact that PH::GFP is known to bind specifically to plasma membrane-enriched phosphoinositides and does not appear to label internal cell membranes [20], these observations suggest that the invaginations are membrane tubes pulled from the plasma membrane.

We analyzed the dynamic properties of invaginations in live embryos. The invaginations extended up to 12 µm toward the centrosome (mean 2.3±1.8 µm, *n* = 290), and lasted up to 25 s (mean 2.5±2.7 s, *n* = 275) (fig. S4A). Invaginations displayed alternate growing and shrinking phases (fig. S4). For invaginations whose lifetime exceeded 3 s, the mean inward speed was 0.91±0.66 µm/s (*n* = 50) (fig. S4B,C). The outward retraction speed of the invaginations was 1.02±0.86 µm/s (*n* = 61). Interestingly, these velocities are similar to those of both spindle poles following laser cutting (fig. S2C, [7]) and spindle pole fragments following laser-induced centrosome disintegration [8]. These similarities are consistent with invaginations being pulled by the same cortical force generators that drive spindle displacement.

**Figure 2:**
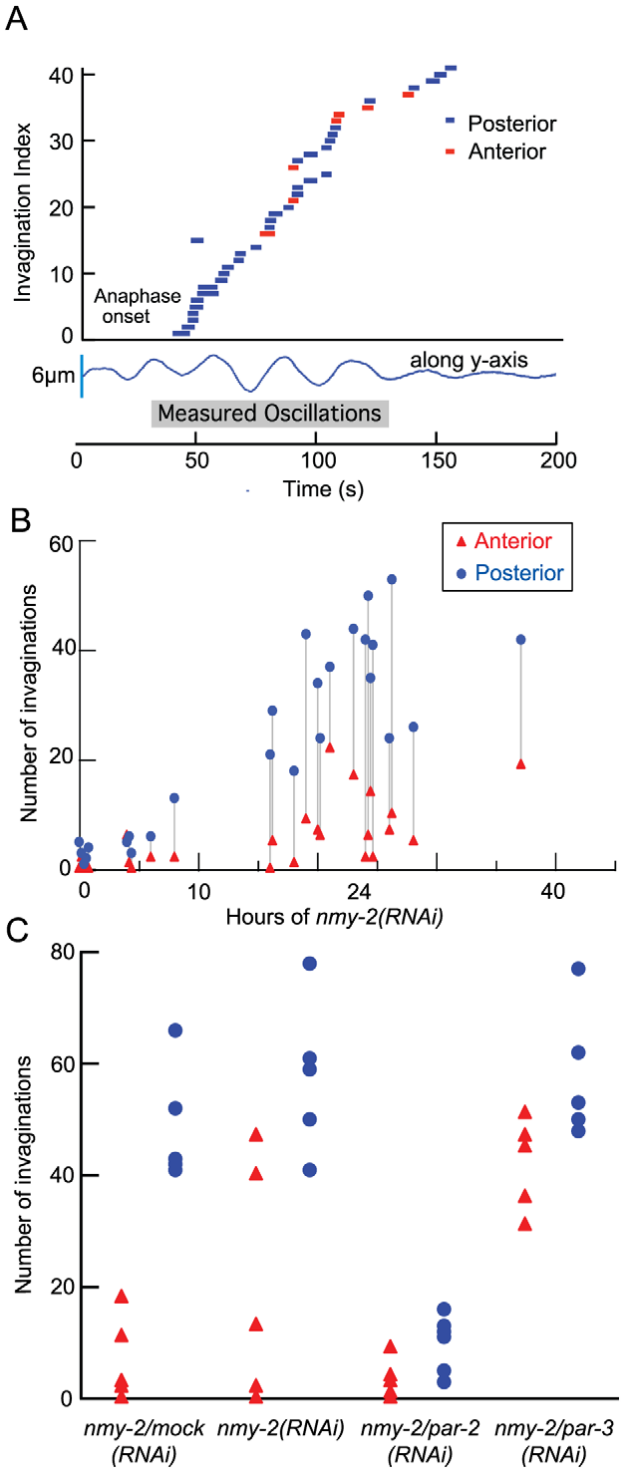
The number of invaginations depends on time following anaphase onset, location in the embryo and PAR-proteins. **A**, Each dash represents an invagination (numbered in order of appearance on the ordinate axis), either formed on the posterior (blue) or anterior pole (red). The length of the dash indicates the duration of the invagination. The y-axis shows the index of the invagination, the x-axis gives the time in seconds. Onset of anaphase corresponds approximately to the start of the transverse oscillations shown underneath in blue. **B**, Number of invaginations in either the posterior (blue dot) or the anterior (red triangle) half of the embryo during anaphase increases with increasing duration of *nmy-2 (RNAi)* treatment. Grey lines pair the data coming from individual embryos. **C**, Comparison of invaginations formed in either the posterior (blue dot) or the anterior (red triangle) half of the embryo after 24 h of double *nmy-2/mock(RNAi)*, 24 h of *nmy-2 (RNAi)* and 24 h of double *par-2/nmy-2 (RNAi)* respectively *par-3/nmy-2 (RNAi)*; for corresponding values, see Fig. 3. Both in *par-2/nmy-2 (RNAi)* and *par-3/nmy-2 (RNAi)*, no significant difference could be found between anterior and posterior count of invaginations.

We next investigated whether the characteristics of invaginations are dependent on the level of NMY-2 depletion. We could not detect any significant dependence (fig. S6): the lifetime and maximal length of the invaginations were similar across depletion times. First, this suggests that once formed, the properties of the invaginations are due to the underlying physical properties of the lipid membrane rather than the particular characteristics of the associated acto-myosin cortex [21]. Second, it suggests that little interaction exists between invaginations, as their properties do not seem to depend on their number, which increases upon NMY-2 run down (fig. 2, fig. S6).

**Figure 3:**
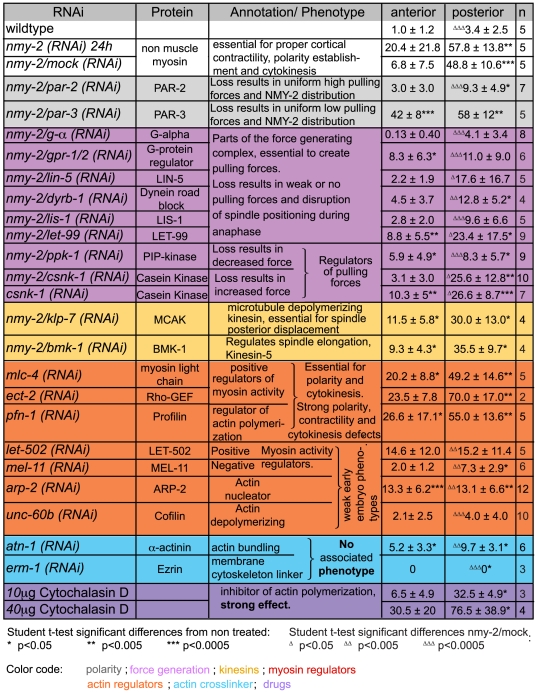
Summary of invagination phenotypes for various genes involved in acto-myosin cortical organization and spindle pulling forces. Invaginations were counted during the whole anaphase on the anterior and posterior pole under different RNAi conditions (mean ± Standard Deviation). Significant difference to control embryos for the anterior and posterior pole is indicated by black asterisks. Significant differences to *nmy-2 (RNAi)* or (*nmy-2/mock (RNAi)* in the case of double RNAi) for the posterior pole are indicated by open triangles. Proteins are classified by their proposed role, indicated by color shading. Grey for proteins involved in polarity, purple for force generation, yellow for kinesins, orange for actin and myosin regulators and turquoise actin crosslinkers.

To demonstrate that these invaginations resulted from a disruption of the acto-myosin cortex and were not specific to the reduction in NMY-2 activity, we examined embryos in which the cortex was disrupted pharmacologically. To avoid inhibiting polarity establishment, we added cytochalasin D to embryos at the beginning of mitosis. This treatment led to similar numbers of invaginations when compared to *nmy-2 (RNAi)* (32.5±4.9, *n* = 3) (fig. 1A, third panel, fig. S7, video S3, fig. 3), suggesting that invaginations are due to a general weakening of the acto-myosin cortex. Consistent with this conclusion, we found that RNAi of several key effectors of acto-myosin function in the one-cell embryo also led to large numbers of invaginations. This included ECT-2, which, among its potential roles, is required for normal activation of myosin contractility [22], [23], [24], MLC-4, a myosin regulatory light chain essential for NMY-2 activity [13], and profilin (PFN-1), which is essential for the cortical actin network [25], [26] (fig. 3). Because strong depletion of any of these proteins results in polarity establishment defects, loss of cortical contractility and cytokinesis failure, we used partial depletion conditions under which PAR domains were able to form by the onset of anaphase. Depletion of components that have relatively minor or no phenotype in the one-cell embryo led to a moderate or no increase in the number of invaginations. These include ARP-2, the Rho kinase LET-502, the myosin phosphatase MEL-11 [27], cofilin (UNC-60b), the actin cross-linker alpha-actinin (ATN-1), and ERM-1, which has been proposed to couple the acto-myosin cortex to the overlying membrane [28], [29]. The depletion of ARP2, LET-502, MEL-11, UNC-60b or ATN-1 did lead to a small increase in the number of invaginations, suggesting that invaginations are sensitive to subtle defects in the acto-myosin cortex. However, we did not find a clear pattern in the degree to which depletion of these proteins led to invaginations and thus could not identify specific aspects of the acto-myosin cortex, for example, the degree of cross-linking or turnover, that are particularly critical for resisting the formation of invaginations. Thus, at this point, we can only conclude that invaginations reflect a general defect in the function of the acto-myosin cortical meshwork.

**Figure 4:**
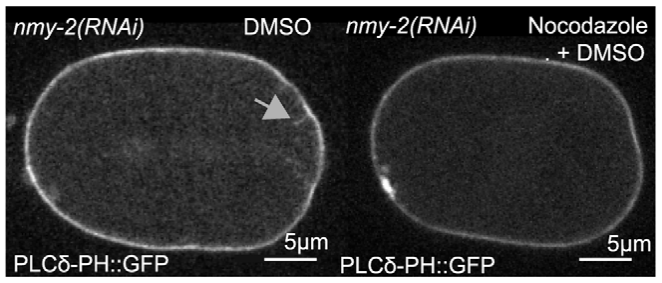
Invaginations require microtubules. Left panel shows a *nmy-2 (RNAi)* embryo expressing PH::GFP as a membrane marker treated with DMSO. An invagination is marked with a gray arrow. The right panel shows an *nmy-2 (RNAi)* embryo treated with nocodazole dissolved in DMSO: no invaginations were observed (see video S2).

The fact that invaginations occur primarily in the posterior and appear to extend from the membrane towards the centrosome, suggests that the invaginations are caused by microtubule-dependent pulling forces that arise from the cortical force generators involved in spindle positioning. We therefore examined *nmy-2 (RNAi)* embryos expressing PH::GFP and tubulin::mCherry. Although, due to imaging limitations, we could not directly identify microtubules attached to each invagination, we found several instances where an invagination was associated with a prominent astral microtubule (fig. S2A,B). To confirm that these invaginations were microtubule dependent, we treated *nmy-2 (RNAi)* embryos with nocodazole, a microtubule depolymerizing drug, which eliminated invaginations (0 invaginations measured in *n* = 14 embryos, fig. 4, Video S3). We could also induce invaginations during anaphase with cytochalasin D and then subsequently suppress the resulting invaginations by treatment with nocodazole (video S3). Finally, we found that laser cutting of the spindle, which relieves astral microtubule-dependent pulling forces, eliminated invaginations (data not shown). Together these data indicate that invaginations require microtubule-dependent pulling forces.

Besides the posterior localization and microtubule-dependence of the invaginations, several other lines of evidence support a correlation between cortical force generators and the appearance of invaginations. First, the number of invaginations depended on the cell cycle: the number of invaginations increased between metaphase (0 invaginations both on anterior and posterior halves, in *n = 17* embryos) and anaphase (29±25 and 51±18, in *n = 4* embryos, respectively) (fig. 2A). This increase parallels the increase in the activity of the cortical force generators during the cell cycle [30], [31]. Second, depletion of either *par-2* or *par-*3, which leads to loss of embryonic polarity and symmetric pulling forces [7], when combined with RNAi of *nmy-2*, led to an equal number of invaginations on the posterior and anterior cortices (fig. 2C). Consistent with *par-2 (RNAi)* leading to “anteriorization” of the embryo and uniformly low pulling forces [7], *par-2*/*nmy-2 (RNAi)* embryos exhibited relatively few invaginations. Conversely, when we combined *par-3 (RNAi)*, which leads to “posterization” of the embryo and uniformly high pulling forces [7], with *nmy-2 (RNAi)*, we obtained high numbers of invaginations. Thus, the position and magnitude of the invaginations correlates with the pattern of cortical force generator activity in embryos whose polarity has been perturbed.

Cortical force generation in the *C. elegans* embryo is regulated by a G protein pathway [8], [32]. We analyzed embryos subjected to double RNAi, targeting *nmy-2* in combination with components of this pathway that are essential for the generation of pulling forces [32]: *goa-1*/*gpa-16* encode two partially redundant G-protein Gα subunits and are involved in the positive regulation of pulling forces; *gpr-1* and *gpr-2*, which are 97% identical and encode partially redundant GDP dissociation inhibitors, which also positively regulate pulling forces; and, *lin-5*, which encodes a putative actin-binding protein. All double RNAi experiments gave the same result: the number of invaginations was significantly reduced compared to *nmy-2/mock (RNAi)* controls, particularly on the posterior pole (fig. 3, fig. S3). Similarly, fewer invaginations were seen after RNAi of dynein or dynactin subunits, such as *dyrb-1*, which is a subunit of the dynein complex and is required for the generation of normal pulling forces [33] (fig. 3, fig. S3). Together, these results indicate that the formation of invaginations is dependent on the G-protein-dependent force-generating machinery responsible for driving spindle displacement.

The force generating complexes may be attached to the membrane through myristoyl groups on the G-proteins [34], [35]. We asked whether the invaginations contain additional cortical components. We analyzed co-localization of NMY-2 and PAR-2 with invaginations, using worms expressing NMY-2::GFP with either PAR-2::cherry or PH::cherry, after treatment with *pfn-1 (RNAi)* or *mlc-4 (RNAi)*. We found that the invaginations co-localized with foci of NMY-2 in some, but not all cases (fig. S1A), and often contained the polarity protein PAR-2 (fig. S1A, video S1). Thus, the invaginations contain cortical components, although it is unclear whether they are simply associated with the invaginating membrane by virtue of their localization or are also associated with the force generating complex (see below).

While a direct correlation between invaginations and cortical force generator activity is attractive, we considered an alternative hypothesis for the asymmetry in invaginations, i.e. more on the posterior side than on the anterior side. In polarized embryos, acto-myosin cortex distribution is asymmetric, with higher concentrations of myosin occurring within an anterior cap and lower concentrations in the posterior. Therefore, in principle, one could imagine that the asymmetry of the invaginations could arise from differences in acto-myosin-dependent cortical rigidity in the anterior and posterior, rather than differences in force generator activity. This would be consistent with the model of Kozlowski *et al.* [15], who postulated that the softer posterior cortex prevents detachment of cortical force generators and allows for more sustained pulling forces. However, when we examined embryos treated with cytochalasin D, which disrupts the acto-myosin cortex in both hemispheres (fig S7), we still obtained posterior spindle displacement and an unequal number of invaginations on the posterior and anterior poles (fig. 3). Similarly, in *mlc-4 (RNAi)*, we found that NMY-2 asymmetry was lost, but invaginations remained asymmetric (fig. S7, fig. 3). Finally, in *nmy-2 (RNAi)* where levels of NMY-2 are barely above background fluorescence and no clear asymmetries are visible, again invaginations are asymmetric. Notably, in all three cases, pulling forces remain asymmetric as spindles underwent normal posterior displacement (fig. S7, fig. 3). Together these data suggest that both the distribution of invaginations and the asymmetry in pulling forces reflects the distribution of active force generators rather than asymmetries in the stiffness of the acto-myosin cortex.

We have shown that weakening the acto-myosin cortex increases the number of invaginations. We asked whether the number of invaginations could also be increased by increasing cortical pulling forces, even when the cortex was unperturbed. CSNK-1, the *C. elegans* casein kinase, together with PPK-1, a PI(4)P5-kinase, transduce PAR-polarity cues into asymmetric Gα regulation. PPK-increases pulling forces, and CSNK-1 decreases them by inhibiting the localization of PPK-1 to the cortex [36]. We found that RNAi of *ppk-1* in combination with *nmy-2* reduced the number of invaginations 5-fold compared to *nmy-2 (RNAi)* alone. This is similar to the reduction caused by double *gpr-1/2, nmy-2 (RNAi)* (fig. 3) and consistent with PPK-1 being a positive regulator of force generation. Interestingly, depletion of CSNK-1, which relieves the inhibition of PPK-1 and results in abnormally high pulling forces [36], led to large numbers of invaginations even in the presence of a normal cortex (fig. S5 A,B and fig. 3). Thus, increasing cortical force generation alone is sufficient to increase the number of invaginations. When cortical force generation was increased by RNAi of *csnk-1* in embryos whose cortex was also weakened by *nmy-2 (RNAi)*, no further increase in the number of invaginations was observed (fig. 3). This suggests that invaginations seen in *nmy-2 (RNAi)* and *csnk-1 (RNAi)* are due to the same force generators, and that all the force generators that are engaged produce an invagination.

## Discussion

The existence of asymmetric microtubule-dependent pulling forces in the one-cell embryo is well established. However, visualization of the sites of force generation on the cortex has remained elusive. Here, we have described the formation of plasma membrane invaginations that are pulled by astral microtubules in a process that reflects the distribution of spindle pulling forces. The cell cycle variation in their abundance, their localization within the cell, and the sensitivity of their distribution to depletion of components with known roles in generating asymmetric spindle pulling forces, provide strong evidence that the invaginations are pulled by the same force-generating machinery that leads to asymmetric spindle positioning. Thus, the invaginations mark the sites of the force generators.

The ability to visualize sites of cortical force generation provides us with the opportunity to examine the number and distribution of force generators in the embryo. Previous work based on analysis of the movement of centrosome fragments suggested that the number of active force generators, was ∼100, with about two-fold more on the posterior side [8]. Our present results, which are based on an entirely independent methodology, suggest a strikingly similar picture: By extrapolating the number of invaginations seen in the medial plane to the whole posterior surface area (see material and methods), we estimate a total of 80 simultaneous invagination sites on the posterior side at the end of anaphase. This suggests that there are at least 80 cortical force generators on the posterior side. Because the number of invaginations is lower on the anterior side, our result suggests that the number of force generators is smaller in the anterior half of the embryo. Although both analyses require particular assumptions, in our case that each active force generator tends to create one invagination, we find it satisfying that these two completely independent methods arrive at a similar picture regarding the number and distribution of cortical force generators.

The formation of invaginations in the *csnk-1 (RNAi)* may explain why spindle movements following CSNK-1 depletion are not faster, as might be expected if the net force is larger. The invaginations would tend to decrease the forces and so tend to bring the net force on the spindle back to wildtype levels.

What is the mechanical basis underlying the formation of invaginations? The invaginations are likely to be thin tubes of membrane and associated protein with a diameter of about 200 nm or less. Their ability to retract suggests that they are still connected to the cortex and their homogeneous fluorescence indicates that their diameter remains roughly constant. It is formally possible that these invaginations could reflect some form of failed endocytosis events. However, the spatial and temporal patterns of their appearance, as well as their dependency on the force generation machinery seem inconsistent with such a model. We also failed to observe co-localization with a marker of early endosomes, EEA-1. If the invaginations are a form of endocytosis, then it is an unconventional one.

Theoretical and *in vitro* studies [18], [19], [21], [37] have estimated that pulling a tube from a cell or a giant liposome requires a force exceeding a threshold that depends on the tension and the bending rigidity of the membrane. For a tube with a diameter of 200 nm and the tension and bending stiffness of *in vivo* lipid membrane, the force is about 8 pN. This force is in the range of the maximum force that can be generated by a single motor protein or a depolymerizing microtubule [38]. Thus, the invaginations could be pulled by a force-generating element that contains a small number of motor proteins. If the cortical proteins increase the net rigidity of the cell membrane beyond that of lipid alone, the required number of motors in the force generating element would be proportionally higher. The threshold force required to create an invagination is determined by the mechanical properties of the membrane and cortex. Because the properties of invaginations, such as their length and duration did not change substantially following NMY-2 rundown, even though their number changed significantly, it appears that the properties of the force generators such as speed and processivity are not altered when the mechanical properties of the cortex are altered. Consistent with this idea, we observed less than one hundred invaginations at any given time, most of them being a few microns long, which means that the expansion of the cytoplasmic membrane area remains below 2%. As a consequence, the cytoplasmic membrane, modeled as a fluid, stays in the entropic elasticity regime and the membrane tension — and thus the force required to pull an invagination — is not increased by the presence of other invaginations [21], [37], [39].

It has been proposed that during rapid pulling of membrane tubes, frictional forces arising from the coupling of the acto-myosin cortex to the membrane, lead to a broadening of the tube at its base [40], [41]. It is possible that the broad bases of the invaginations that we observe by SPIM (fig. 1B) arise from this effect. If the cortex is mechanically rigid or forms a fine meshwork, then it will oppose broadening and it will be more difficult to form an invagination. If perturbation of the acto-myosin cortex leads to a weakening or coarsening of the meshwork, then invaginations would be expected to form at lower forces. However, because we are currently unable to visualize this meshwork in the vicinity of invaginations, we hesitate to draw a firm conclusion in this respect.

In summary, the invaginations we describe provide insight into the localization and distribution of the sites of force generation in C. *elegans* embryos and suggest a model for the transduction of cortical force generation into spindle movement. Cortical force generators, which our results suggest are likely anchored in the membrane, exert force on the spindle by pulling on microtubules. If the membrane is sufficiently strong and stiff due to the supporting effects of the acto-myosin cortical network, the spindle moves toward the cortex. If this rigidity is compromised or pulling forces excessively strong, the membrane moves instead toward the spindle, forming an invagination. Our data confirm that asymmetric spindle positioning is due to the asymmetric localization of a moderate number of force generators that are active at the end of anaphase.

## Materials and Methods

### Culturing *C. elegans*

*C. elegans* embryos were cultured as described in [42]. All strains were maintained at 16°C and shifted to 20–25°C for imaging. The transgenes encoding the GFP, YFP or mCherry fusion proteins were under the control of the *pie-1* promotor. Transgenic worms were created by microparticle bombardment (BioRad), as described [43].

### *C. elegans* strains used

N2 (wildtype); TH120 (GFP::Par-2;mCherry::Par-6) [44]; OD58 (PH::GFP(PLC1δ1) [16]; JA1559 (mCherry:: tubulin, Ahringer Lab); OD70 (PH::mCherry) [45]]; JJ1473 (NMY-2::GFP) [46]], TH155 (mCherry:: tubulin (JA1559)/PH::GFP (OD70)). To generate TH220 (*unc-119*(ed3); *pie-1::lifeact::gfp*) the coding sequence for the LifeAct probe [47] was codon optimized in JCat (www.jcat.de) - 5′ ATGGGAGTTGCTGATCTTATTAAA AAATTCGAATCTATTTCTAAAGAAGAA-3′ - cloned into pTH304 (gift from Andrei Pozniakovsky) and introduced by microparticle bombardment as described [43]. The following dual labeled combinations were generated through standard genetic crosses: TH155/TH120 (GFP::Par2/mCherry:: tubulin), JJ1473/OD70 (NMY-2::GFP/mCherry:: tubulin).

### Gene silencing by RNA interference

RNAi experiments were performed by feeding as described by Timmons *et al.* [48]. In the case of double RNAi experiments (*lin-5 (RNAi)/nmy-2 (RNAi), gpr-1/2 (RNAi)/nmy-2 (RNAi), par2 (RNAi)/nmy-2 (RNAi)*, *par3 (RNAi)/nmy-2 (RNAi), ppk-1/nmy-2 (RNAi) and rsa-1/nmy-2 (RNAi)*) co-injection of dsRNA was performed [49]. *G-alpha (RNAi)/nmy-2 (RNAi), let-99/nmy-2 (RNAi), csnk-1/nmy-2 (RNAi), lis-1/nmy-2 (RNAi), dyrb-1/nmy-2 (RNAi), klp-7/nmy-2 (RNAi) and bmk-1/nmy-2 (RNAi)* were performed by co-feeding. Worms were grown for 4–30 h (according to the individual dsRNA) at 25°C on feeding plates, or on normal plates after injection. Spe-1 (defective spermatogenesis) was used as *mock (RNAi)*.

### Feeding clones

Target genes were subcloned into the RNAi feeding vector L4440 and transformed into HT115 bacteria. The feeding clones for *f08f8.2*, *pfn-1*, *arx-2*, *mel-11*, *let-502* and *erm-1* were ordered from Gene Service. The feeding clone for *mlc-4* was constructed by cloning genomic DNA into pL4440-dest-RNAi [50]. *ect-2* and *csnk-1* were obtained from the Ahringer lab, for *g-alpha* see [51]. The feeding clone for *unc-60b* (cofilin) was a gift from Carrie Cowan, the feeding clone for *dyrb-1* was a gift from Monica Gotta.

### dsRNAi production

A region from the target gene was amplified in a PCR reaction, using N2 genomic DNA as a template. The PCR-sample was purified afterwards using the Qiagen PCR cleanup Kit.

For T3 and T7 transcription, the Ambion kit was used. The sample was purified using the RNeasy kit. Primers used to amplify regions from N2 genomic DNA are listed in the table:

### *Gene* Primer sequence

*nmy-2* TAATACGACTCACTATAGGAATTGAATCTCGGTTGAAGGAA **T7**

AATTAACCCTCACTAAAGGACTGCATTTCACGCATCTTATG **T3**

*par-2* TAATACGACTCACTATAGGACCTCTGCCCAAATTTTCAA **T7**

AATTAACCCTCACTAAAGGTCTCAAAACTCGGCCACATA **T3**

*par-3* TAATACGACTCACTATAGGGTGACCGGACGTGAAACTG **T7**

AATTAACCCTCACTAAAGGTTTTCCTTCCGAGACCTTCC **T3**

*gpr-1* TAATACGACTCACTATAGGTCAGCGGTTGTTTTATTGAAGAT **T7**

AATTAACCCTCACTAAAGGTGGACGAGCTGGAAAAATATAAA **T3**

*lin-5* TAATACGACTCACTATAGGCGAGCAAAGAAGTCTGGAGG **T3**

AATTAACCCTCACTAAAGGCGTTCCTCTCTTCGTCAAGG **T7**

*atn-1* TAATACGACTCACTATAGGATCGAGGGTGTCAAGTTGG **T3**

AATTAACCCTCACTAAAGGCTCTCTGGAGGTGGAGCAAC **T7**

The remaining primer sequences for dsRNAs were taken from the Cenix screen [52], the sequences of which are available at www.wormbase.org.

### Invaginations Analysis

Confocal images of embryos were obtained at approximately 20°C with an Olympus IX71 spinning disc set up using a 100×/NA 1.35 Oil Iris UPlan Apochromat objective and Yokogawa scan head CSU10. 488 nm and 561 nm lasers were used for illumination. Images were acquired with an Andor Ixon EMCCD 512×512 camera at 250 ms exposure with no binning. Andor IQ was used as acquisition software. Embryos were dissected in M9 buffer and mounted with 2% agarose pads. Image processing was performed with ImageJ. The number of invaginations was counted manually. Error bars and statistical significance of invagination counting were calculated using the Poisson distribution [53].

For selective plane illumination microscopy (SPIM)), PH::GFP embryos were prepared in 0.5% agarose gel, and imaged using an objective 63× NA 1.0, excitation and observation wavelength respectively of 488 nm and 510 nm.

### Spindle cutting experiments

The laser ablation experiments were performed using a highly focused ultraviolet laser beam and observed using differential interference contrast (DIC) and spinning-disk microscopy as described in [8]. All ablations were performed through DIC optics. Embryos were dissected in M9 buffer and mounted with 2% Agarose pads. The spindle was cut in the middle just after anaphase onset (identified by the disappearance of the metaphase plate) and observed by DIC microscopy. Five to ten shots (57 pulses, 470 Hz) were fired at the middle of the spindle.

### Nocodazole treatment

The laser ablation experiments were performed as above, except that a Melles Griot Argon-Ion Laser (488 nm, 100 mW) laser was used for fluorescence imaging. All ablations were performed in DIC optics. A hole was shot in the eggshell just after anaphase onset (identified by the disappearance of the metaphase plate) and observed by DIC microscopy. Five to ten shots (each of 57 pulses, 470 Hz) were fired at the edge of the eggshell. If the same number of shots was fired in the region between spindle and cortex, no hole formed and no phenotype was observed. After shooting the eggshell, the embryos were visualized at 488 nm. Embryos were dissected and mounted on Poly-Lysine coated slides using a buffer consisting of 1 part M9/2 parts 0.1 M NaCl +4% Sucrose. The nocodazole concentration was 5 mg/ml in DMSO for the stock, diluted 1∶500 in buffer.

### Cytochalasin D treatment

Embryos were rendered permeable by *F08F8.2 (RNAi)*, which results in osmo-sensitivity that can be rescued by growth in osmotically balanced media [54]. Embryos were dissected and mounted in SGM (Shelton's embryonic growth media [13]) to maintain osmotic balance, and cytochalasin D was added to a final concentration of 10 µg/ml either at the time of pronuclear meeting or metaphase. A detailed description will appear elsewhere (NWG, unpublished data).

### Centrosome tracking

TH27 embryos were imaged using an upright microscope (Axiovision Imager 2e, Zeiss, Jena, Germany), modified for long-term timelapse. First, an extra anti-heat filter was added on the mercury lamp light path. Second, to decrease the bleaching and obtain optimal excitation, we used an enhanced transmission 12 nm bandpass excitation filter centered on 485 nm (AHF analysentechnik, Tübingen, Germany). Images were collected by an EMCCD camera (Andor technologies) using Solis software.

Tracking of centrosomes, as well as analysis on trajectories were obtained by a custom tracking software based on [31], and developed using Matlab (The MathWorks). Tracking of 4° C methanol-fixed γTUB::GFP embryos indicated an accuracy of 10 nm.

Except otherwise stated, all values are given in an axis linked to the embryo: the x-axis is the anterior-posterior axis with positive values oriented toward the posterior pole; y-axis was chosen to be perpendicular and centered midway along the x-axis. The axis was centered on the geometric center of the embryo. Embryo orientation and center were obtained by cross-correlation of embryo background cytoplasmic fluorescence with an artificial binary image mimicking an embryo or by contour detection of the cytoplasmic membrane using background fluorescence of cytoplasmic γTUB::GFP with help of an active contour algorithm [55].

Results were averaged over all replicates for each condition. Statistical significance was established using a two-tailed, two samples Students t-test with Welch-Satterthwaite correction for unequal variances.

The local fluorescence of γ-Tubulin::GFP at the midpoint of the two centrosomes was measured. This point lies in the center of the nucleus and therefore an increase in fluorescence is observed at nuclear envelope breakdown (NEBD) [56]. Because of bleaching, the fluorescence is constantly decreasing at all other times. This transient fluorescent increase provided a reliable reference time point and thus all times are relative to NEBD unless otherwise indicated.

### Calculating the number of active force generators

To obtain the number of active force generators, we counted the number of invaginations observed in one plane and extrapolated to obtain an estimate of the total number at each timepoint. We assumed the pole to be a hemisphere with the surface A_pole_. The plane seen in our microscope image has the surface A_plane._ Counting the number of invaginations in this plane, allowed us to estimate the number of invaginations per µm^2^. Assuming that the distribution of invaginations is uniform in the pole hemisphere we were able to estimate the number of active force generators by multiplying A_pole_ by the estimated number of invaginations per µm^2^.

## Supporting Information

Figure S1 **Localization of PAR-2 and NMY-2 to invaginations.** **A**, First row: micrograph of invaginations obtained after *nmy-2 (RNAi)* treatment; PAR-2 is labeled with GFP and tubulin with mCherry. Second row: micrograph of invaginations obtained after *pfn-1 (RNAi)* treatment; PAR-2 is labeled with mCherry and NMY-2 with GFP. Third row: micrograph of invaginations obtained after *mlc-4 (RNAi)*; NMY-2 is tagged with a GFP and a mCherry tags PH domain. Scale bar is 10 µm. **B**, Survival rate of laid eggs is reduced with increasing duration of *nmy-2 (RNAi)* treatment. After 4 h of *nmy-2 (RNAi)* 91.3±13.4% (n = 71; mean ± standard deviation) of the laid eggs hatch, after 6 h of *nmy-2 (RNAi)* 77.8±27.2% (n = 28) hatch, after 12 h of *nmy-2 (RNAi)* 94.3±10.3% (n = 290) eggs hatch, after 16 h of *nmy-2 (RNAi)* 96±9% (n = 242) of the eggs hatch, after 20 h of *nmy-2 (RNAi)* 58.9±29.5% (n = 564) hatch and after 24 h of *nmy-2 (RNAi)* only 30.1±29.4% (n = 496) eggs hatch. **C**, Size of the PAR-2 domain in percentage of the entire embryonic circumference in wild-type and with increasing duration of *nmy-2 (RNAi)* treatment. In wild-type embryos the size of the PAR-2 domain is 51.6±1.7% at metaphase (n = 4; mean ± standard deviation), in embryos treated with *nmy-2(RNAi)* for 22 h 42.5±6.3% (n = 4), after 24 h of *nmy-2 (RNAi)* 29.3±12.7% (n = 11), after 28 h of *nmy-2 (RNAi)* 24.3±18.9% (n = 4) and after 31 h of *nmy-2 (RNAi)* its reduced to 24.3±7.6% (n = 3).
(6.19 MB TIF)

Figure S2 ***nmy-2 (RNAi)*** **does not affect spindle positioning and pulling forces.** **A**, Plot of the position of the posterior centrosome along the anterior posterior axis in γ-tub::GFP embryos (left panel), and *nmy-2 (RNAi)* embryos (right panel). **B**, Schematic of a laser-cutting experiment. The spindle is cut at anaphase onset with an UV-laser-beam. Velocity of the anterior and posterior centrosomes are tracked after the laser-cut and plotted in B. **C**, Laser-cutting experiments in control and *nmy-2 (RNAi)* treated embryos. The velocities of the two centrosomes, anterior (red) and posterior (blue), were measured after the spindle was cut at anaphase onset using an UV-laser beam. In control embryos, the anterior centrosome moved with a velocity of 0.53±0.05 µm/s and the posterior with a velocity of 1.0±0.1 µm/s (n = 4; mean ± SD). After treatment with *nmy-2 (RNAi)* for 24 h, the anterior centrosome moved with a velocity of 0.77±0.11 µm/s after the spindle cut, the posterior centrosome with 0.98±0.07 µm/s (n = 6; mean ± SD).
(0.52 MB TIF)

Figure S3 **Effect of depletion of genes involved in force generation on invaginations.** Box plot of the number of invaginations on the anterior (red) and posterior (blue) pole throughout anaphase in all performed double RNAis. For corresponding values, see Table 1. On each box the central mark indicates the median, the edges of the box are the 25^th^ and 75^th^ percentiles. Dashed line indicates the spreading of data points. Outliers are marked with a +.
(0.38 MB TIF)

Figure S4 **Properties of invaginations in** ***nmy-2 (RNAi)*** **.** **A**, Distribution of the maximum length of invaginations. The median length 1.73 µm is indicated by an arrow. Also shown is the distribution of the lifetime of invaginations, the median lifetime 1.77 s is indicated by an arrow. **B**, Distribution of the average growing and shrinking speed of invaginations. Medians are 0.71 µm/s and 0.74 µm/s respectively and are indicated by arrows. **C**, Trajectories of 5 representative invaginations with a duration ≥3 s. The trajectories start at the second frame in which an invagination is visible. The red line indicates a trajectory with a constant growing speed of 0.71 µm/s (median of growing speed).
(0.19 MB TIF)

Figure S5 ***csnk-1 (RNAi)*** **increases the number of invaginations.** **A**, Embryo treated with *csnk-1 (RNAi)* during centration and rotation of the nuclear centrosome complex. Embryos depleted of CSNK-1 show a stronger formation of invaginations in comparison to embryos treated with *nmy-2 (RNAi)* (see B), indicating higher forces in *csnk-1 (RNAi)* during centering and rotation. **B**, Embryo treated with *nmy-2 (RNAi)* during centration and rotation of the nuclear centrosome complex.
(2.11 MB TIF)

Figure S6 **Properties of invaginations upon a NMY-2 rundown.** Box plot of (**A**) the maximum length, (**B**) the lifetime, (**C**) average growing and (**D**) average shrinking speed of invaginations counted on both anterior and posterior side and obtained upon running down NMY-2. On each box the central mark indicates the median, the edges of the box are the 25^th^ and 75^th^ percentiles. Dashed line indicates the spreading of data points. Outliers are marked with a +. Only the growth rate significantly depends on level of *nmy-2 (RNAi)* (one-way anova p = 0.003). Each group contains 3 embryos. (**E**) Number of invaginations upon running down NMY-2; each diamond is a single embryo. Distribution of the characteristics of invaginations (**F-I**) after 4-6 h, (**J-M**) after 16-20 h, (**N-Q**) after 24-28 h of *nmy-2 (RNAi)*. Distribution of (**F, J, N**) the maximum length of invaginations, (**G, K, O**) the lifetime of invaginations, (**H, L, P**) the average growing and (**I, M, Q**) shrinking speed of invaginations. Color shading encodes the rundown time groups.
(1.19 MB TIF)

Figure S7 **Localization of NMY-2 and actin upon cortex weakening.** **A**, Localization of NMY-2::GFP during anaphase and in wild-type embryos (top) and after 24 h of *mlc-4 (RNAi)* (bottom). Scale bar is 5 µm. **B** LifeAct::GFP expressing embryo, subjected to *f08f8.2 (RNAi)* before (top) and after treatment with cytochalasin D (bottom). Scale bar is 5 µm
(2.87 MB TIF)

Video S1 Formation of invaginations in an embryo labeled with PAR-2::GFP and PAR-6::cherry after 23 h of *nmy-2 (RNAi)* treatment. Frames were collected every 20 seconds, the display rate is 10 frames per second (200× real time).
(4.32 MB MOV)

Video S2 Formation of invaginations in an embryo labeled with PH::GFP after 23 h of *nmy-2 (RNAi)* treatment. Frames were collected every 3 seconds, the display rate is 10 frames per second (30× real time).
(0.35 MB MOV)

Video S3 Formation of invaginations in an embryo labeled with PLCδ-PH::GFP after Cytochalasin D treatment. PH::GFP, *f08f8.1(RNAi)* embryo was dissected in SGM (Shelton growth medium) and allowed to reach anaphase. The addition of Cytochalasin D during anaphase leads to large numbers if invaginations which can be suppressed by the subsequent addition of Nocodazole in addition to Cytochalasin D. 5 s intervals, 10 frame per second (50× real time).
(0.75 MB MOV)
