# Supplementary figures and images for: Membrane Invaginations Reveal Cortical Sites that Pull on Mitotic Spindles in One-Cell C. elegans Embryos
Source: PLoS One. 2010 Aug 20;5(8):e12301. doi: 10.1371/journal.pone.0012301 (PMC2924899; doi:10.1371/journal.pone.0012301)

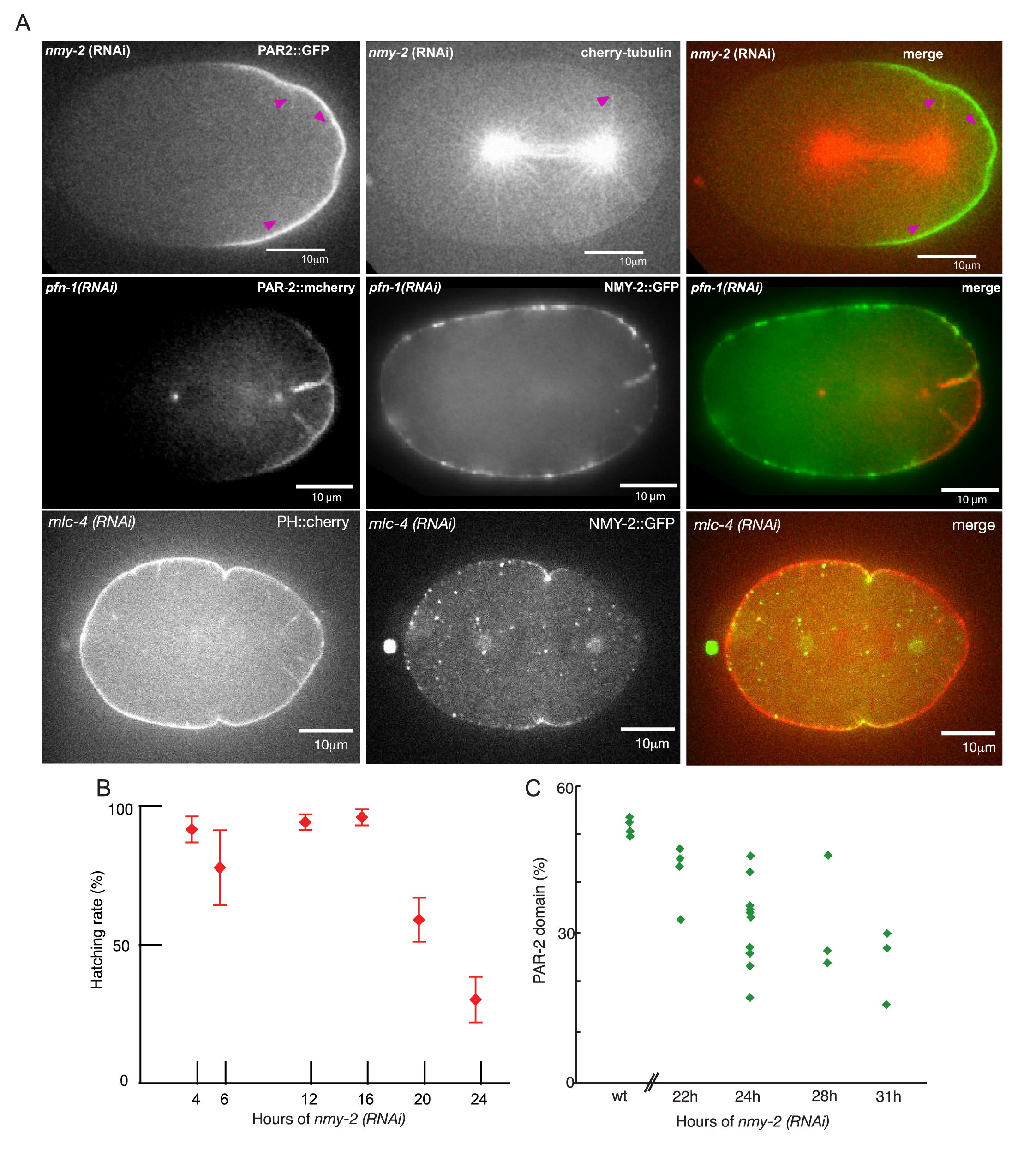

Supplement: Figure S1 — Localization of PAR-2 and NMY-2 to invaginations. A, First row: micrograph of invaginations obtained after nmy-2 (RNAi) treatment; PAR-2 is labeled with GFP and tubulin with mCherry. Second row: micrograph of invaginations obtained after pfn-1 (RNAi) treatment; PAR-2 is labeled with mCherry and NMY-2 with GFP. Third row: micrograph of invaginations obtained after mlc-4 (RNAi); NMY-2 is tagged with a GFP and a mCherry tags PH domain. Scale bar is 10 µm. B, Survival rate of laid eggs is reduced with increasing duration of nmy-2 (RNAi) treatment. After 4 h of nmy-2 (RNAi) 91.3±13.4% (n = 71; mean ± standard deviation) of the laid eggs hatch, after 6 h of nmy-2 (RNAi) 77.8±27.2% (n = 28) hatch, after 12 h of nmy-2 (RNAi) 94.3±10.3% (n = 290) eggs hatch, after 16 h of nmy-2 (RNAi) 96±9% (n = 242) of the eggs hatch, after 20 h of nmy-2 (RNAi) 58.9±29.5% (n = 564) hatch and after 24 h of nmy-2 (RNAi) only 30.1±29.4% (n = 496) eggs hatch. C, Size of the PAR-2 domain in percentage of the entire embryonic circumference in wild-type and with increasing duration of nmy-2 (RNAi) treatment. In wild-type embryos the size of the PAR-2 domain is 51.6±1.7% at metaphase (n = 4; mean ± standard deviation), in embryos treated with nmy-2(RNAi) for 22 h 42.5±6.3% (n = 4), after 24 h of nmy-2 (RNAi) 29.3±12.7% (n = 11), after 28 h of nmy-2 (RNAi) 24.3±18.9% (n = 4) and after 31 h of nmy-2 (RNAi) its reduced to 24.3±7.6% (n = 3). (6.19 MB TIF) [file pone.0012301.s001.tif]

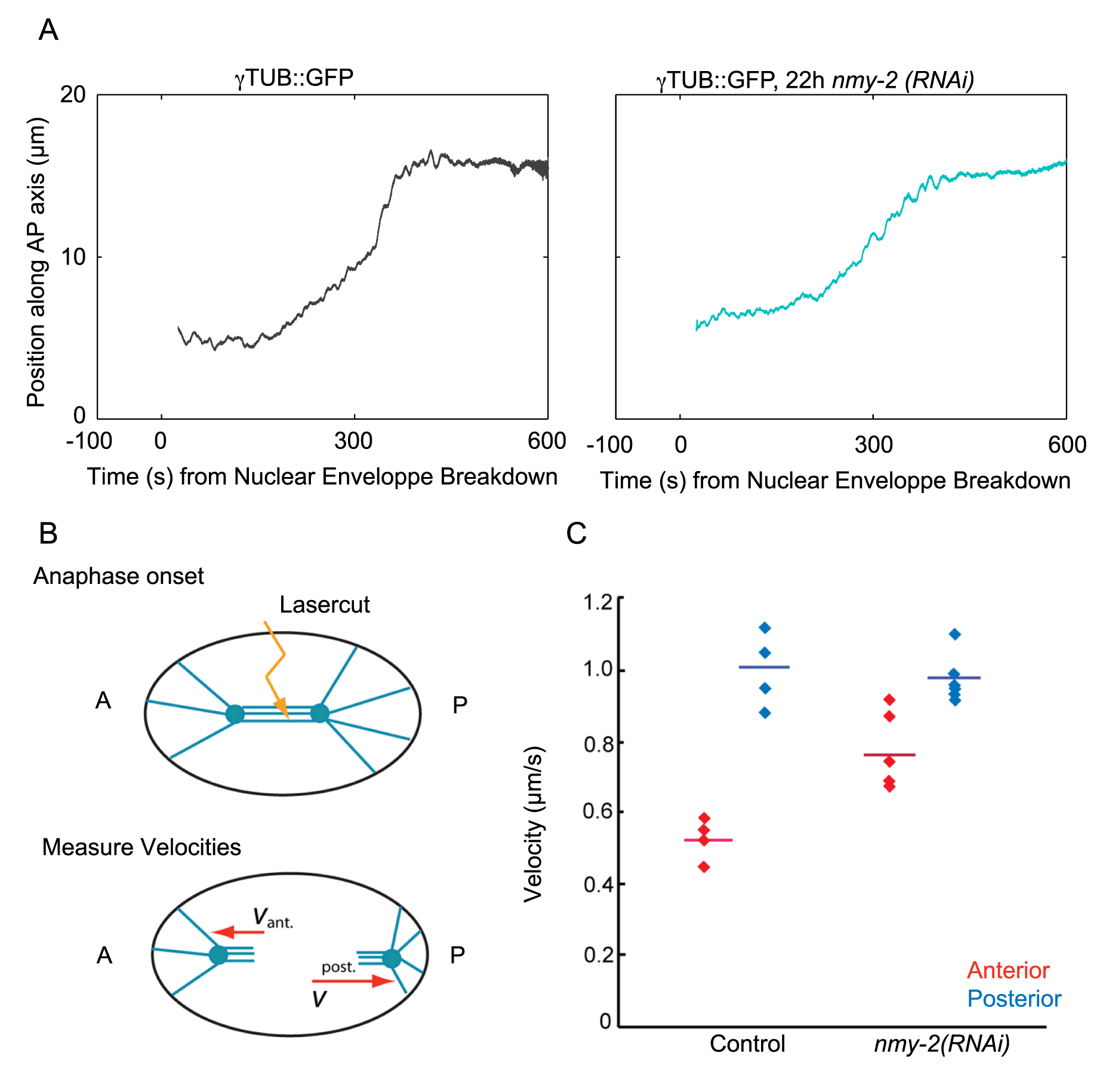

Supplement: Figure S2 — nmy-2 (RNAi) does not affect spindle positioning and pulling forces. A, Plot of the position of the posterior centrosome along the anterior posterior axis in γ-tub::GFP embryos (left panel), and nmy-2 (RNAi) embryos (right panel). B, Schematic of a laser-cutting experiment. The spindle is cut at anaphase onset with an UV-laser-beam. Velocity of the anterior and posterior centrosomes are tracked after the laser-cut and plotted in B. C, Laser-cutting experiments in control and nmy-2 (RNAi) treated embryos. The velocities of the two centrosomes, anterior (red) and posterior (blue), were measured after the spindle was cut at anaphase onset using an UV-laser beam. In control embryos, the anterior centrosome moved with a velocity of 0.53±0.05 µm/s and the posterior with a velocity of 1.0±0.1 µm/s (n = 4; mean ± SD). After treatment with nmy-2 (RNAi) for 24 h, the anterior centrosome moved with a velocity of 0.77±0.11 µm/s after the spindle cut, the posterior centrosome with 0.98±0.07 µm/s (n = 6; mean ± SD). (0.52 MB TIF) [file pone.0012301.s002.tif]

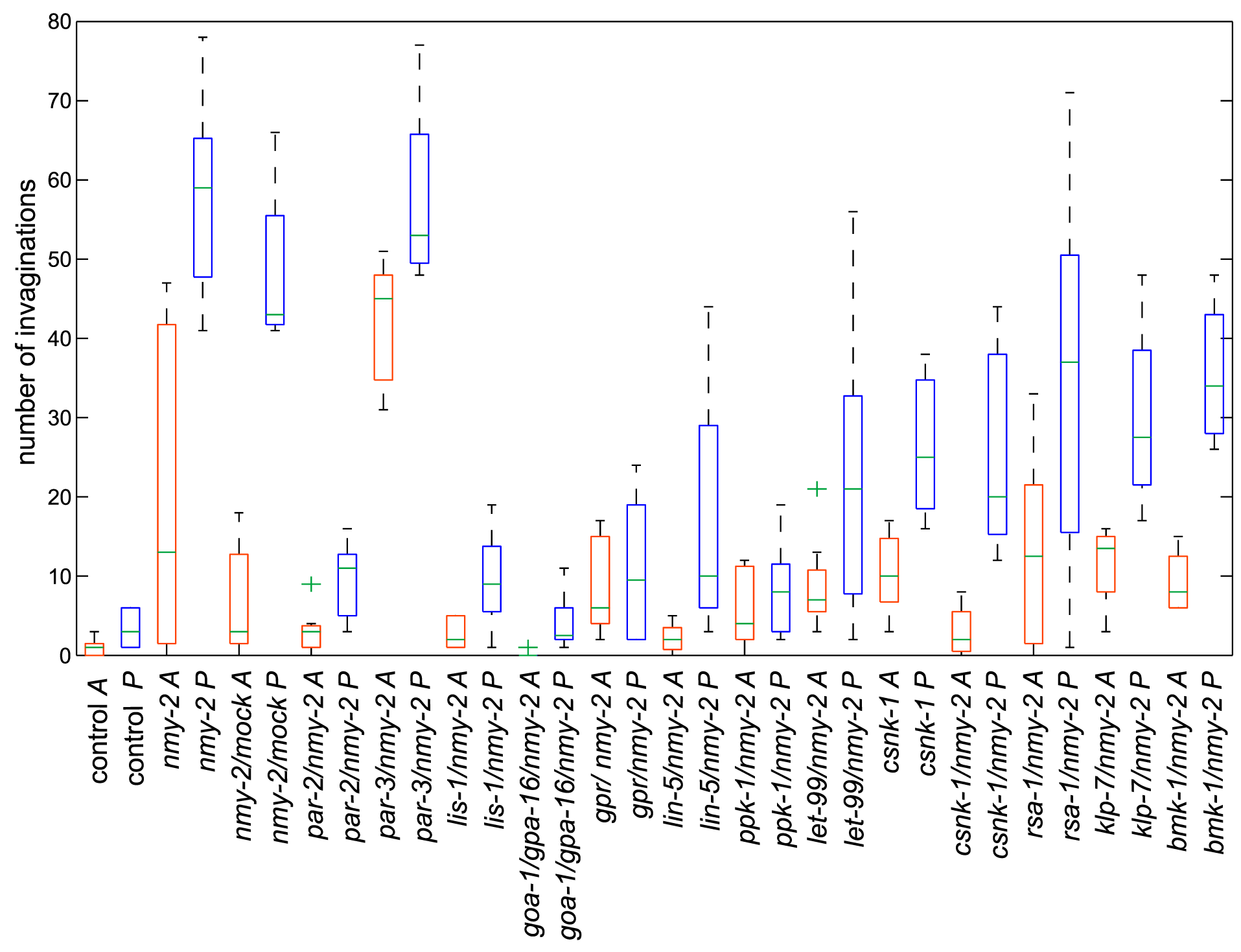

Supplement: Figure S3 — Effect of depletion of genes involved in force generation on invaginations. Box plot of the number of invaginations on the anterior (red) and posterior (blue) pole throughout anaphase in all performed double RNAis. For corresponding values, see Table 1. On each box the central mark indicates the median, the edges of the box are the 25th and 75th percentiles. Dashed line indicates the spreading of data points. Outliers are marked with a +. (0.38 MB TIF) [file pone.0012301.s003.tif]

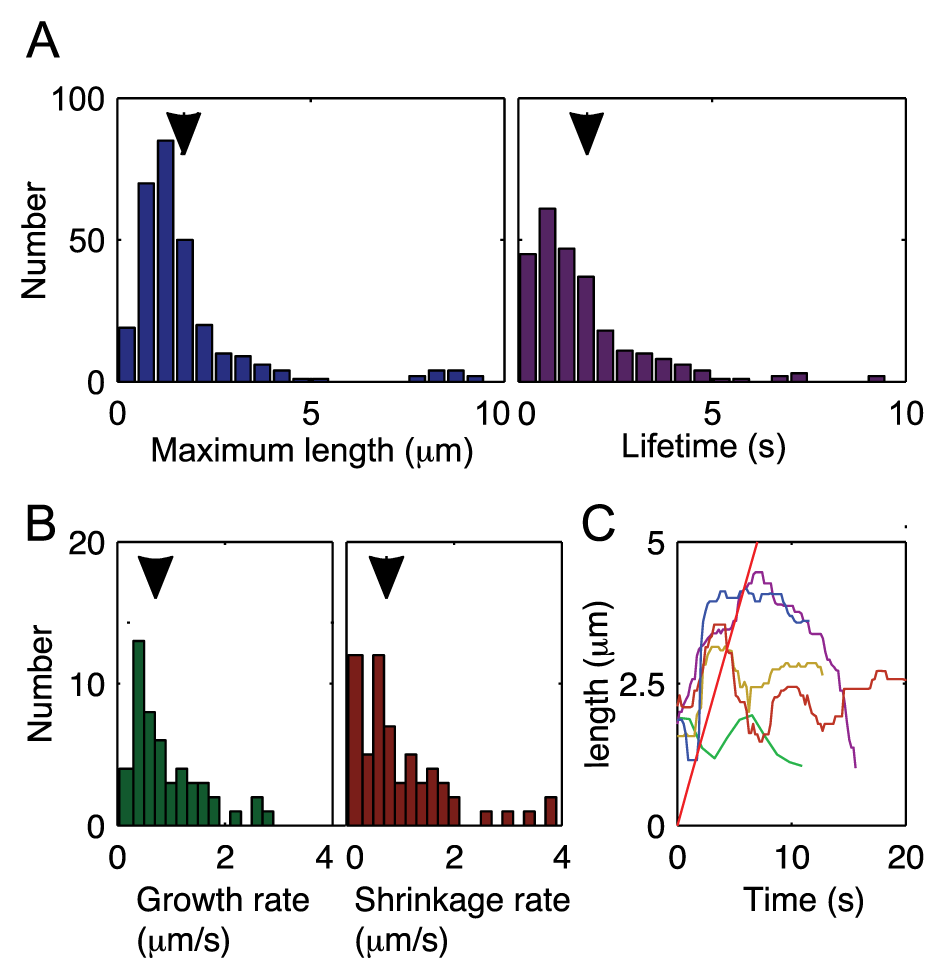

Supplement: Figure S4 — Properties of invaginations in nmy-2 (RNAi) . A, Distribution of the maximum length of invaginations. The median length 1.73 µm is indicated by an arrow. Also shown is the distribution of the lifetime of invaginations, the median lifetime 1.77 s is indicated by an arrow. B, Distribution of the average growing and shrinking speed of invaginations. Medians are 0.71 µm/s and 0.74 µm/s respectively and are indicated by arrows. C, Trajectories of 5 representative invaginations with a duration ≥3 s. The trajectories start at the second frame in which an invagination is visible. The red line indicates a trajectory with a constant growing speed of 0.71 µm/s (median of growing speed). (0.19 MB TIF) [file pone.0012301.s004.tif]

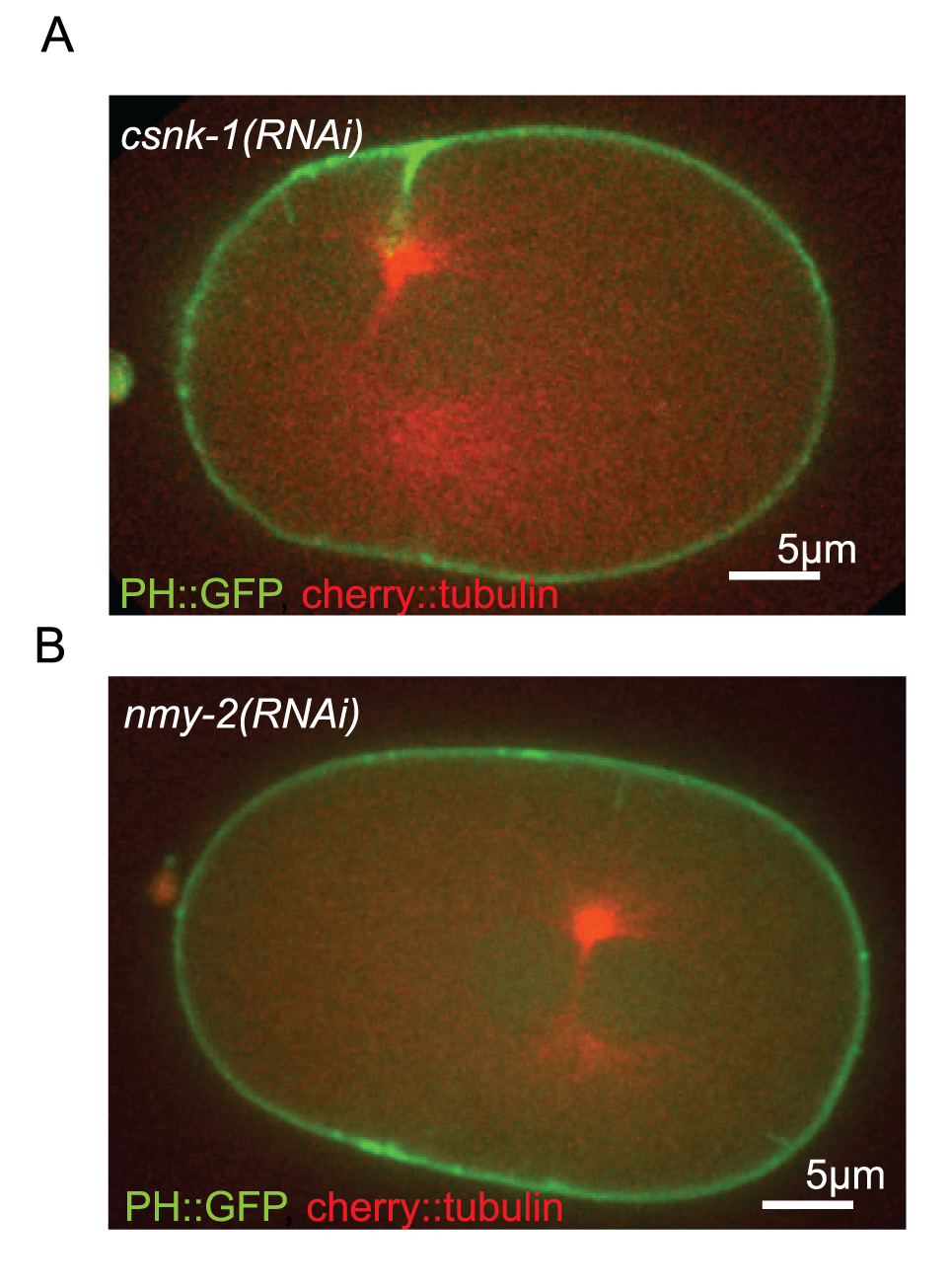

Supplement: Figure S5 — csnk-1 (RNAi) increases the number of invaginations. A, Embryo treated with csnk-1 (RNAi) during centration and rotation of the nuclear centrosome complex. Embryos depleted of CSNK-1 show a stronger formation of invaginations in comparison to embryos treated with nmy-2 (RNAi) (see B), indicating higher forces in csnk-1 (RNAi) during centering and rotation. B, Embryo treated with nmy-2 (RNAi) during centration and rotation of the nuclear centrosome complex. (2.11 MB TIF) [file pone.0012301.s005.tif]

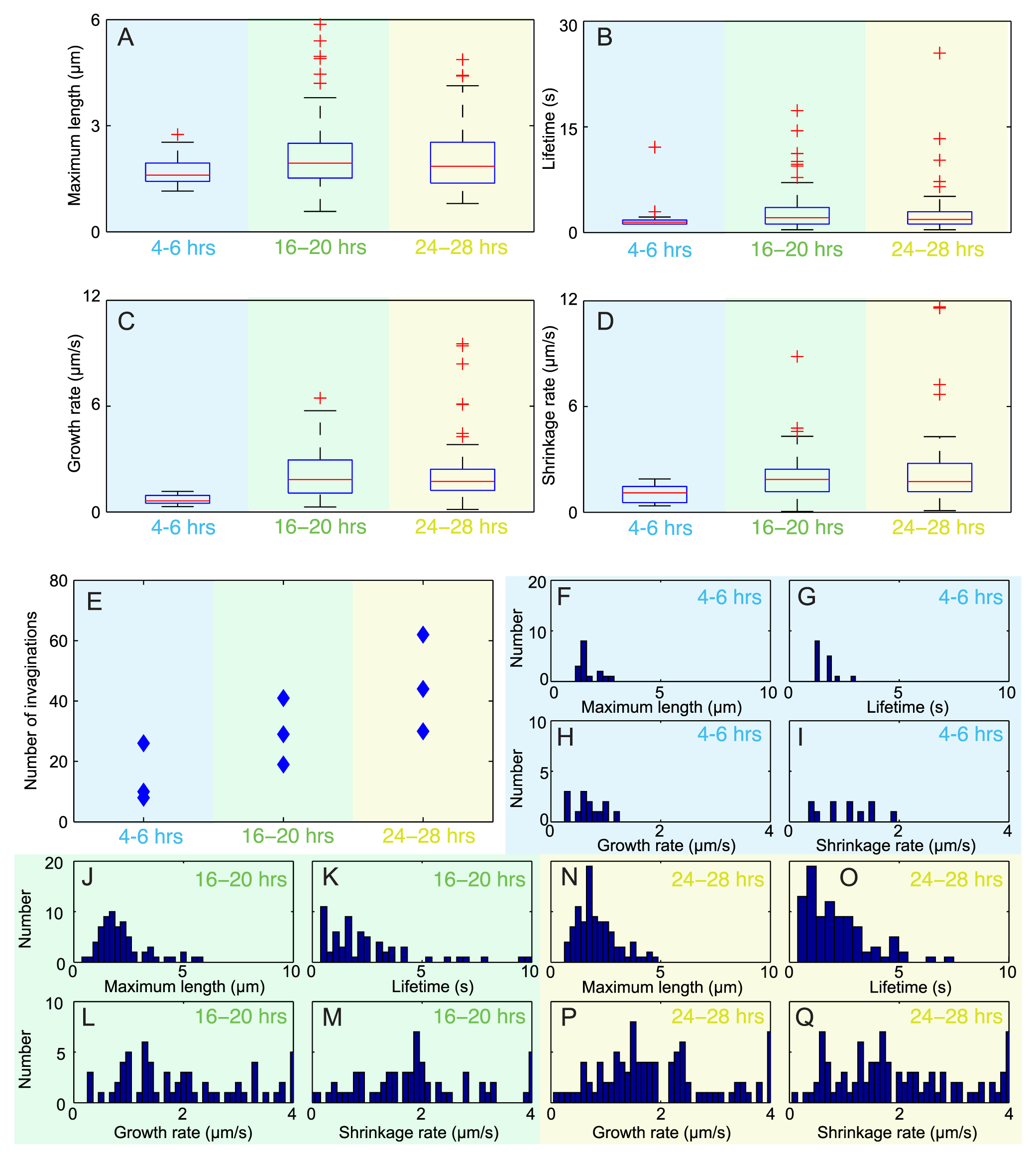

Supplement: Figure S6 — Properties of invaginations upon a NMY-2 rundown. Box plot of (A) the maximum length, (B) the lifetime, (C) average growing and (D) average shrinking speed of invaginations counted on both anterior and posterior side and obtained upon running down NMY-2. On each box the central mark indicates the median, the edges of the box are the 25th and 75th percentiles. Dashed line indicates the spreading of data points. Outliers are marked with a +. Only the growth rate significantly depends on level of nmy-2 (RNAi) (one-way anova p = 0.003). Each group contains 3 embryos. (E) Number of invaginations upon running down NMY-2; each diamond is a single embryo. Distribution of the characteristics of invaginations (F-I) after 4-6 h, (J-M) after 16-20 h, (N-Q) after 24-28 h of nmy-2 (RNAi). Distribution of (F, J, N) the maximum length of invaginations, (G, K, O) the lifetime of invaginations, (H, L, P) the average growing and (I, M, Q) shrinking speed of invaginations. Color shading encodes the rundown time groups. (1.19 MB TIF) [file pone.0012301.s006.tif]

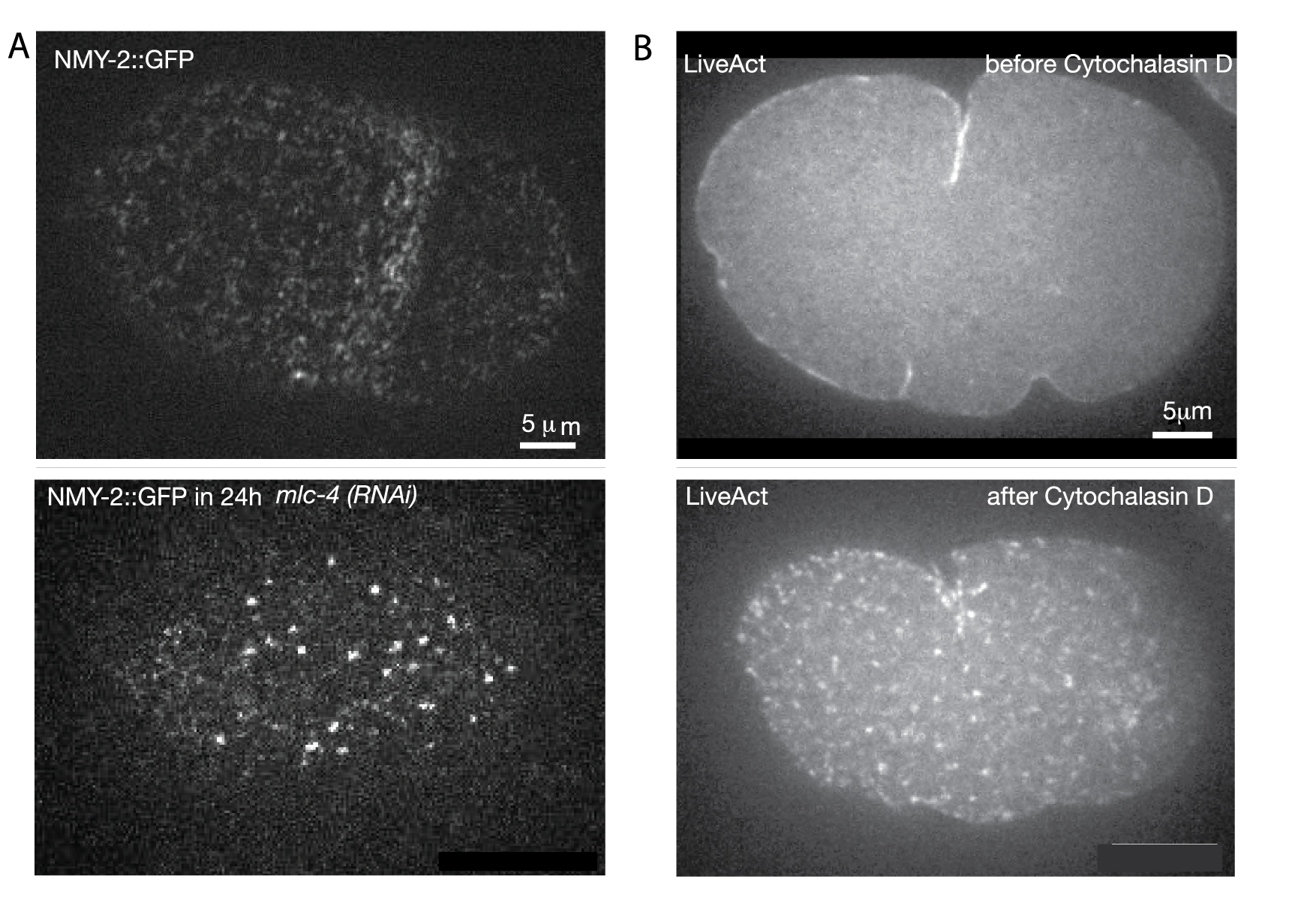

Supplement: Figure S7 — Localization of NMY-2 and actin upon cortex weakening. A, Localization of NMY-2::GFP during anaphase and in wild-type embryos (top) and after 24 h of mlc-4 (RNAi) (bottom). Scale bar is 5 µm. B LifeAct::GFP expressing embryo, subjected to f08f8.2 (RNAi) before (top) and after treatment with cytochalasin D (bottom). Scale bar is 5 µm (2.87 MB TIF) [file pone.0012301.s007.tif]
